# Evaluation of three inflammation-associated blood indices for predicting malignancy in thyroid nodules

**DOI:** 10.20945/2359-4292-2026-0014

**Published:** 2026-02-16

**Authors:** Merve Çatak, Bülent Koca, Zeynep Çetin, Özden Özdemir BAŞER

**Affiliations:** 1 Tokat Gaziosmanpasa University, Faculty of Medicine, Department of Endocrinology and Metabolism, Tokat, Türkiye; 2 Tokat Gaziosmanpasa University, Faculty of Medicine, Department of General Surgery, Tokat, Türkiye; 3 Amasya Sabuncuoğlu Şerefeddin Training and Research Hospital, Amasya Şerefettin Sabuncuoğlu, Faculty of Medicine, Department of Endocrinology and Metabolism, Amasya, Türkiye; 4 Dokuz Eylul University, Faculty of Medicine, Department of Endocrinology and Metabolism, Izmir, Türkiye

**Keywords:** Differentiated thyroid carcinoma, systemic immune-inflammation index, systemic inflammation response index, pan-immune inflammation value, inflammation-based biomarkers, preoperative diagnosis, thyroid nodules

## Abstract

**Objective:**

Differentiated thyroid carcinoma (DTC) is the most common endocrine malignancy and usually has a favorable prognosis. However, both diagnostic and prognostic evaluations currently rely mainly on postoperative histopathological results. Systemic inflammation-based indices — such as the Systemic Immune-Inflammation Index (SII), Systemic Inflammation Response Index (SIRI), and Pan-Immune Inflammation Value (PIV) — have recently emerged as potential biomarkers in various cancers. This study aimed to evaluate the diagnostic and prognostic utility of these indices in patients undergoing thyroid surgery.

**Subjects and methods:**

This retrospective study included 554 patients who underwent total thyroidectomy between 2014 and 2021. Tumors were categorized as benign or malignant according to final histopathology. SII, SIRI, and PIV were calculated from preoperative complete blood counts. Multivariate logistic regression was performed and included age, sex, thyroid-stimulating hormone (TSH) level, glycated hemoglobin (HbA1c) level, and diabetes status. Receiver operating characteristic (ROC) analysis was used to determine diagnostic performance.

**Results:**

Among 554 patients, 366 had benign and 188 had malignant tumors. Among the systemic inflammatory markers, only the SII differed significantly between groups (p = 0.002) and remained an independent predictor of malignancy in multivariate analysis (OR = 0.85 per 100-unit increase, p = 0.007). ROC analysis revealed an AUC of 0.597, with 65.8% sensitivity and 58.2% specificity. None of the indices demonstrated prognostic value in the subgroup analyses.

**Conclusion:**

The SII demonstrated independent but clinically limited diagnostic value in differentiating malignant from benign thyroid lesions. Although its accuracy was poor (AUC <0.6), the SII may serve as a low-cost adjunct within multivariable preoperative models, particularly in indeterminate cytology cases.

## INTRODUCTION

Thyroid cancer is the most common endocrine malignancy worldwide, and its incidence has been steadily increasing in recent decades ^(1)^. The majority of cases are classified as differentiated thyroid carcinoma (DTC), which includes papillary thyroid carcinoma (PTC) and follicular thyroid carcinoma (FTC), both of which are generally associated with a favorable prognosis ^(2)^. While fine-needle aspiration biopsy (FNAB) is the gold standard for the preoperative evaluation of thyroid nodules, prognostic classification typically relies on postoperative histopathological features ^(3,4)^. However, both diagnostic and prognostic assessments in clinical practice currently depend heavily on invasive or postoperative procedures. There is a pressing need for accessible, noninvasive biomarkers that can assist in differentiating benign from malignant disease and support early risk prediction.

Inflammation is recognized as a key player in tumor initiation, progression, and metastasis ^(5)^. In the context of cancer, tumor-associated inflammation involves not only local immune cell infiltration but also systemic immune responses that can be captured via hematologic indices derived from routine blood tests ^(5,6)^. Among these, the Systemic Immune-Inflammation Index (SII) — calculated using neutrophil, lymphocyte, and platelet counts — has demonstrated diagnostic utility and prognostic relevance in several malignancies, including hepatocellular carcinoma and gastrointestinal cancers ^(7,8)^. Similarly, the Systemic Inflammation Response Index (SIRI), which incorporates the levels of neutrophils, monocytes, and lymphocytes, has emerged as an alternative composite marker reflecting systemic immune balance ^(9)^.

More recently, the Pan-Immune Inflammation Value (PIV) has been introduced as a comprehensive biomarker that integrates four key circulating immune components: neutrophils, lymphocytes, platelets, and monocytes ^(10)^. Unlike traditional markers such as the neutrophil-to-lymphocyte ratio (NLR), these multiparameter indices aim to provide a broader and more robust representation of the host’s inflammatory and immune status ^(10)^. These indices represent simple, reproducible, and cost-effective markers that can be readily derived from routine blood tests, which enhances their potential clinical applicability. In addition to being associated with cancer, the SII, SIRI, and PIV have been shown to be associated with disease activity in autoimmune, cardiovascular, and other chronic inflammatory conditions, highlighting their potential clinical versatility ^(11-13)^.

Although systemic inflammation indices have been investigated in patients with thyroid malignancies, most studies have focused primarily on the SII, whereas direct head-to-head comparisons of the SII, SIRI, and PIV in the same cohort remain scarce. Therefore, the incremental value of simultaneously evaluating these three indices under uniform clinical conditions has not been fully established ^(14)^.

Given the established link between chronic inflammation and tumorigenesis, metabolic conditions such as diabetes mellitus deserve particular attention. Diabetes mellitus is a chronic low-grade inflammatory condition that can alter systemic immune and metabolic pathways. Recent studies have shown that elevated inflammatory indices are associated with increased mortality in patients with type 2 diabetes, reflecting a sustained proinflammatory state ^(15)^. Moreover, diabetes itself has been identified as a potential risk factor for thyroid cancer, possibly through mechanisms involving insulin resistance, hyperinsulinemia, and chronic inflammation ^(16)^. Therefore, glucose and HbA1c levels were also assessed in our study to account for the potential metabolic and inflammatory effects of diabetes on systemic inflammation indices.

Although several studies have explored the prognostic utility of these indices in thyroid cancer, their diagnostic value — particularly in distinguishing benign from malignant nodules in the preoperative setting — is unclear. Therefore, this study was designed to assess both the diagnostic utility and potential prognostic significance of the SII, SIRI, and PIV in patients undergoing thyroid surgery.

## SUBJECTS AND METHODS

This retrospective study was conducted at Tokat Gaziosmanpasa University Faculty of Medicine and included patients who underwent total thyroidectomy between January 2014 and December 2021. This study was approved by the Tokat Gaziosmanpasa University Clinical Research Ethics Committee (approval number: 21-KAEK-135) and was conducted in accordance with the principles of the Declaration of Helsinki. All patient data were anonymized to ensure confidentiality.

### Patient selection

Patients aged 18 years or older with available preoperative laboratory results and postoperative histopathological diagnoses were considered eligible for inclusion. Indications for thyroidectomy included Bethesda IV–VI cytology results, Bethesda III nodules with suspicious ultrasound features or repeated Bethesda III results, nodules larger than 4 cm, nodules causing compressive symptoms, and hyperfunctioning nodules (toxic multinodular goiter or toxic adenoma).

The exclusion criteria included a history of autoimmune thyroid diseases (such as Graves’ disease and Hashimoto’s thyroiditis or histopathological evidence of thyroiditis in the nontumoral thyroid parenchyma), pregnancy, rheumatologic or hematologic disorders, corticosteroid or immunosuppressive therapy, active infection, chronic liver or kidney disease, the presence of nonthyroidal malignancy, and incomplete laboratory data. Additionally, patients who were diagnosed with medullary or anaplastic thyroid carcinoma, noninvasive follicular thyroid neoplasms with papillary-like nuclear features (NIFTPs), tumors of uncertain malignant potential, or toxic nodular disease were excluded.

**Figure 1:**
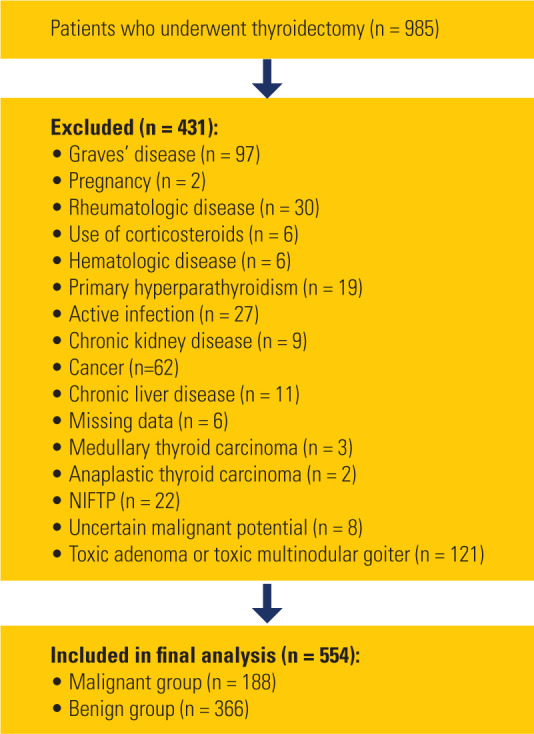
Patient selection flowchart.

A flowchart showing the patient selection process is presented in **Figure 1**.

### Demographic and clinical data

Demographic information, including age and sex, was recorded for all included patients. On the basis of histopathological examination of the thyroidectomy specimens, patients were categorized into two groups: the benign group, consisting of individuals with nonneoplastic thyroid lesions, and the DTC group, consisting of patients diagnosed with papillary or follicular thyroid carcinoma.

### Surgical and histopathological evaluation

Total thyroidectomy was performed as the standard surgical approach for all patients. In cases where preoperative cervical ultrasonography revealed pathological lymphadenopathy, either central or lateral neck lymph node dissection was performed according to clinical guidelines. In the DTC group, detailed histopathological features, including tumor size, histological subtype, capsular and vascular invasion, extrathyroidal extension, perineural invasion, and the presence of lymph node or distant metastases, were recorded. Tumor staging was conducted according to the 8th edition of the American Joint Committee on Cancer (AJCC) staging system ^(17)^.

### Laboratory analysis

All laboratory data were obtained from fasting blood samples collected within one week before surgery. Since all patients were scheduled for elective operations, none had acute illness at the time of sampling. CBC parameters — including neutrophil, lymphocyte, monocyte, and platelet counts — were measured using a Sysmex XN-Series hematology analyzer (Sysmex Corp., Kobe, Japan). Fasting blood glucose levels were measured using the hexokinase enzymatic method, and HbA1c levels were assessed by high-performance liquid chromatography (HPLC). All analyses were performed in institutional biochemistry and hematology laboratories using standardized protocols.

### Inflammatory index calculations

The following systemic inflammatory indices were calculated from the preoperative CBC parameters:

- **Systemic Immune-Inflammation Index (SII):** (neutrophil count × platelet count)/lymphocyte count.
- **Systemic Inflammation Response Index (SIRI):** (neutrophil count × monocyte count)/lymphocyte count.
- **Pan-Immune Inflammation Value (PIV):** (neutrophil count × monocyte count × platelet count)/lymphocyte count.

### Statistical analyses

All the statistical analyses were performed using IBM SPSS Statistics for Windows, version 25.0 (IBM Corp., Armonk, NY, USA). Continuous variables are presented as the mean ± standard deviation (SD) for normally distributed data or as the median (minimum–maximum) for nonnormally distributed data, while categorical variables are expressed as frequencies and percentages.

The distribution of continuous variables was assessed using the Kolmogorov–Smirnov and Shapiro–Wilk tests. For comparisons between the benign and DTC groups, the independent samples *t* test was used for normally distributed variables, and the Mann–Whitney U test was used for nonnormally distributed variables. Categorical variables were compared using Pearson’s chi-square or Fisher’s exact tests, as appropriate.

To evaluate the associations of systemic inflammatory indices (SII, SIRI, and PIV) with thyroid malignancy, univariate logistic regression analyses were initially performed. Variables with a p value < 0.10 in univariate analyses were included in multivariate logistic regression models using a backward stepwise method to identify independent predictors. The results are reported as odds ratios (ORs) with 95% confidence intervals (CIs).

In the DTC group, potential associations between inflammation indices and tumor-related features (such as tumor size, lymph node status, and disease stage) were further assessed using Pearson’s or Spearman’s correlation tests, depending on the data distribution.

Receiver operating characteristic (ROC) curve analysis was performed to evaluate the diagnostic performance of the systemic inflammation-based indices. The area under the curve (AUC), sensitivity, specificity, and optimal cutoff values (determined by the Youden index) are reported for indices that demonstrated statistically significant group differences.

A p value < 0.05 was considered to indicate statistical significance for all analyses.

## RESULTS

A total of 985 patients who underwent thyroidectomy between January 2014 and December 2021 were initially screened. After the exclusion criteria were applied (as illustrated in **Figure 1**), 431 patients were excluded, resulting in a final cohort of 554 patients. These patients were categorized into two groups on the basis of postoperative histopathological findings: the malignant group (n = 188) and the benign group (n = 366). The mean age of the study population was 47.1 ± 11.9 years, with a female-to-male ratio of approximately 4:1.

Comparisons of demographic and laboratory characteristics between the two groups are presented in **Table 1**. The malignant group had a significantly younger mean age than the benign group did (45.10 ± 12.74 vs. 48.45 ± 11.15 years, p < 0.001). No statistically significant difference was observed in sex distribution (p = 0.204). Although the prevalence of diabetes mellitus was greater in the malignant group (13.3%) than in the benign group (10.7%), the difference was not statistically significant (p = 0.151). Serum TSH levels were significantly elevated in patients with malignancy compared with those with benign pathology (1.98 ± 2.07 vs. 1.55 ± 1.30 mIU/L, p = 0.009). No significant differences were observed in free T4, fasting glucose, or HbA1c levels.

**Table 1.** Comparison of demographic and laboratory features between benign and malignant groups

Parameter	Benign group (n = 366)	Malignant group (n = 188)	p-value
Age (years)	48.45 ± 11.15	45.10 ± 12.74	**<0.001**
Sex (F/M)	287/79 (78.4/21.6%)	156/32 (83.0/17.0%)	0.204
DM presence (%)	39 (10.7%)	25 (13.3%)	0.151
TSH (mIU/L)	1.55 ± 1.30	1.98 ± 2.07	**0.009**
Free T4 (ng/dL)	1.22 ± 0.20	1.23 ± 0.22	0.392
Fasting glucose (mg/dL)	112.29 ± 31.40	115.28 ± 39.68	0.108
HbA1c (%)	5.98 ± 1.04	6.15 ± 1.26	0.134
SII	547.08 ± 302.05	654.75 ± 403.30	**0.002**
SIRI	1.13 ± 0.72	1.19 ± 0.86	0.438
PIV	309.91 ± 211.43	331.84 ± 265.21	0.218

Note: Values are presented as mean ± standard deviation or number (percentage), where appropriate.

F/M: female/male; DM: diabetes mellitus; TSH: thyroid-stimulating hormone; Free T4: free thyroxine; HbA1c: glycated hemoglobin; SII: systemic ımmune-ınflammation ındex; SIRI: systemic ınflammation response ındex; PIV: pan-ımmune-ınflammation value.

The tumor characteristics of the malignant group are summarized in **Table 2**. The vast majority had papillary thyroid carcinoma (PTC, 95.2%), and the most common histological variant was classic PTC (59.8%), followed by follicular variant (25.7%), oncocytic (8.9%), and others (5.6%). Multifocal tumors were present in 43.6% of the patients, and bilateral involvement was observed in 33%. The mean tumor diameter was 16.54 ± 13.85 mm, and tumors < 1 cm in diameter were observed in 51.1% of the patients. Capsular invasion (29.8%), extrathyroidal extension (26.6%), and lymph node metastasis (13.8%) were relatively common, whereas vascular invasion (6.9%) and distant metastasis (4.0%) were less common. Radioactive iodine (RAI) therapy was administered to 43.6% of the patients.

**Table 2.** Tumor characteristics in the malignant group

Tumor characteristics	Results
Papillary	179 (95.2%)
Follicular type	9 (4.8%)
PTC variant	
Classic	107 (59.8%)
Follicular	46 (25.7%)
Oncocytic	16 (8.9%)
Others	10 (5.6%)
Number of foci (Mean ± SD)	1.84 ± 1.26
Multifocality	
Yes	82 (43.6%)
No	106 (56.4%)
Laterality	
Unilateral	125 (66.5%)
Bilateral	62 (33.0%)
Tumor size (mm, Mean ± SD)	16.54 ± 13.85
Tumor Size Group	
< 1 cm	96 (51.1%)
1–4 cm	18 (9.6%)
> 4 cm	74 (39.4%)
Capsular invasion	
Yes	56 (29.8%)
No	132 (70.2%)
Lymph node metastasis	
Yes	26 (13.8%)
No	162 (86.2%)
Extrathyroidal extension	
Yes	50 (26.6%)
No	138 (73.4%)
Vascular invasion	
Yes	13 (6.9%)
No	175 (93.1%)
Distant metastasis	
Yes	7 (4.0%)
No	168 (96.0%)
RAI therapy	
Received	75 (43.6%)
Not received	97 (56.4%)

PTC: papillary thyroid carcinoma; RAI: radioactive ıodine; SD: standard deviation.

With respect to preoperative inflammatory markers, the SII was significantly greater in the malignant group than in the benign group (654.75 ± 403.30 vs. 547.08 ± 302.05, p = 0.002). However, the SIRI and PIV values did not differ significantly between groups (p = 0.438 and p = 0.218, respectively).

Univariate analysis demonstrated that age (p = 0.002), TSH level (p = 0.009), and the SII (p = 0.002) were significantly associated with malignancy. In contrast, no significant associations were found for free T4 (p = 0.392), fasting glucose (p = 0.108), HbA1c (p = 0.134), sex (p = 0.204), or diabetes status (p = 0.151).

To identify independent predictors of malignancy, multivariate logistic regression analysis was conducted, including age, TSH level, SII, HbA1c level, sex, and diabetes status. Among these variables, only the Systemic Immune-Inflammation Index (SII) emerged as a statistically significant independent predictor (p = 0.007). Higher SII values were associated with increased odds of malignancy (OR = 1.002, 95% CI: 1.000–1.003). However, the effect size was minimal, as the odds ratio was very close to 1.0, indicating statistical significance but limited clinical relevance. In contrast, age (p = 0.844), TSH level (p = 0.096), HbA1c level (p = 0.389), sex (p = 0.787), and diabetes status (p = 0.849) were not significantly associated with malignancy according to the multivariate model. A detailed overview of the univariate and multivariate logistic regression results is provided in **Table 3**.

**Table 3.** Univariate and multivariate logistic regression analysis for predictors of malignancy

	Univariate OR (95% CI)	Univariate p-value	Multivariate OR (95% CI)	Multivariate p-value
Age	0.984 (0.969–0.998)	**0.025**	0.996 (0.962–1.032)	0.844
TSH	1.157 (1.038–1.289)	**0.009**	1.201 (0.968–1.491)	0.096
SII	1.001 (1.000–1.002)	**0.007**	1.002 (1.000–1.003)	**0.007**
HbA1c	1.086 (0.884–1.334)	0.446	1.221 (0.775–1.922)	0.389
Sex (F/M)	0.743 (0.464–1.190)	0.216	0.887 (0.371–2.118)	0.787
DM presence	1.273 (0.747–2.170)	0.372	1.112 (0.374–3.307)	0.849

OR: odds ratio; CI: confidence ınterval; TSH: thyroid-stimulating hormone; SII: systemic ımmune-ınflammation ındex; HbA1c: glycated hemoglobin; DM: diabetes mellitus; F/M: female/male.

To address the potential overlap between TSH and the SII, additional multicollinearity analyses were performed. The variance inflation factor (VIF) and tolerance values were within acceptable ranges (all VIF < 2.5, tolerance > 0.4), indicating no concerning collinearity. Furthermore, we compared two logistic regression models: In Model 1 (including age, sex, TSH level, HbA1c level, and diabetes status), none of the variables were significant predictors of malignancy. In Model 2 (Model 1 + SII), the SII remained an independent predictor (OR = 0.85 per 100-unit increase, p = 0.007), whereas the TSH level lost statistical significance (p = 0.096). These findings suggest that the predictive effect of the SII is independent of the TSH level.

**Figure 2:**
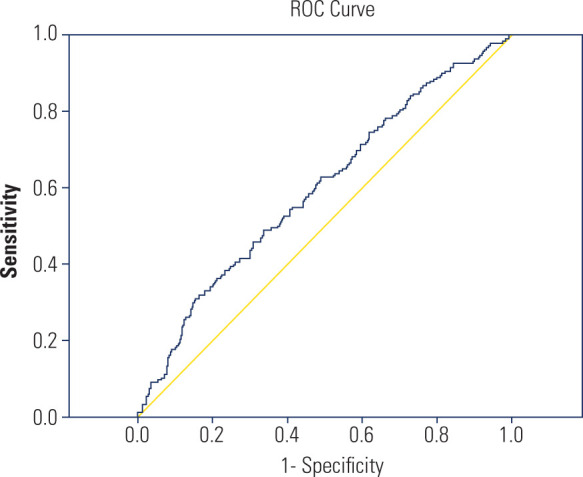
Receiver operating characteristic (ROC) curve of the Systemic Immune-Inflammation Index (SII) for predicting thyroid malignancy. The AUC was 0.597, indicating limited but measurable discriminative ability (cut-off: 487; sensitivity: 65.8%; specificity: 58.2%).

Receiver operating characteristic (ROC) curve analysis was performed to evaluate the diagnostic performance of the Systemic Immune-Inflammation Index (SII) in distinguishing malignant from benign thyroid lesions. The optimal cutoff value for the SII was determined to be 487, with a sensitivity of 65.8% and a specificity of 58.2%. The area under the curve (AUC) was 0.597, indicating limited but statistically measurable discriminatory capacity. The area under the curve (AUC) was 0.597, indicating poor diagnostic accuracy despite statistical significance. The ROC curve of the SII is presented in **Figure 2** to illustrate these findings.

In contrast, neither the Systemic Inflammation Response Index (SIRI) nor the Pan-Immune Inflammation Value (PIV) differed significantly between the benign and malignant groups; therefore, ROC curve analysis was not applicable for these indices.

In addition to evaluating their diagnostic utility, we also assessed the potential prognostic value of preoperative inflammatory indices, including the SII, SIRI, and PIV, within the malignant group. Specifically, their associations with established prognostic parameters such as tumor size (continuous and categorical), multifocality, laterality, lymph node metastasis, extrathyroidal extension, capsular invasion, vascular invasion, and distant metastasis were analyzed. However, all subgroup analyses yielded negative results, indicating that none of the indices demonstrated prognostic utility in relation to these histopathological features.

## DISCUSSION

In this retrospective study, we assessed the diagnostic value of three systemic inflammatory indices — the Systemic Immune-Inflammation Index (SII), Systemic Inflammation Response Index (SIRI), and Pan-Immune Inflammation Value (PIV) — for differentiating malignant from benign thyroid nodules. Among 554 patients, only the SII was significantly associated with malignancy in both the univariate and multivariate analyses, although the effect size was very small (OR: 0.85 per 100-unit increase; 95% CI: 0.75–0.96; p = 0.007), indicating that the clinical impact of this association is limited. In addition, compared with patients with benign nodules, patients with malignant nodules were significantly younger and had higher serum TSH levels, further supporting the known clinical patterns associated with thyroid cancer risk.

The interplay between inflammation and cancer has been the focus of intense research, with increasing evidence implicating the immune system in the initiation, progression, and prognosis of malignancies. The tumor microenvironment, which is composed of immune cells such as neutrophils, lymphocytes, and monocytes, influences tumor behavior through intricate mechanisms involving cytokines, chemokines, and transcription factors ^(5,18,19)^. Chronic inflammation contributes to genetic instability, immune evasion, angiogenesis, and metastasis ^(5)^. Consequently, systemic inflammatory markers derived from routine blood counts — such as the SII, SIRI, and PIV — have gained attention as potential surrogates for tumor–immune interactions in various cancers ^(20,21)^.

Several prior studies have explored these indices in the context of thyroid cancer. For instance, a cohort study demonstrated significantly elevated SII values in patients with DTC compared with those in healthy controls ^(22)^. Similarly, Vural and cols. found higher SII values in DTC patients than in those with multinodular goiter and lymphocytic thyroiditis, suggesting a possible role in distinguishing malignant from benign nodular disease ^(23)^. Zhao and cols. reported that the SII independently predicted lateral lymph node metastasis in papillary thyroid carcinoma (PTC) ^(24)^, whereas Pang and cols. employed machine learning algorithms to emphasize the predictive value of the SIRI for central lymph node metastasis ^(25)^. PIV has also been linked to distant metastasis and higher ATA risk categories in select cohorts ^(26)^.

Although the SII emerged as the only index independently associated with malignancy in our cohort, its overall diagnostic power was limited (AUC = 0.597; sensitivity, 65.8%; specificity, 58.2%), indicating that the SII cannot be considered a standalone diagnostic tool. Instead, it may function as a low-cost adjunct within multivariable preoperative models.

In contrast, evidence from previous studies suggests that for DTC, PIV may have more utility in prognostication than in diagnosis. In a cohort of 376 patients, Öztürk and cols. reported that elevated PIV was significantly associated with distant metastasis (AUC = 0.774), advanced TNM stage (OR > 7), and high ATA risk (OR > 29) ^(26)^. These findings suggest that PIV may reflect more aggressive tumor biology and could serve as a valuable prognostic marker in DTC. However, the inclusion of multiple immune cell types in the PIV formula — particularly monocytes — may introduce heterogeneity, potentially limiting its diagnostic performance in certain settings. In our study, the absence of a significant association between PIV and malignancy may be due to this complexity or study-specific factors such as cohort composition and exclusion criteria.

One of the key strengths of our study lies in its direct comparison of the SII, SIRI, and PIV under the same clinical conditions. Previous research has generally evaluated these indices in isolation; in contrast, our study provides a side-by-side assessment in a moderately large, single-center cohort (n = 554). While the SII remained statistically significant in the multivariate analysis, its effect size was minimal, and its diagnostic accuracy was poor, indicating limited clinical impact. SIRI and PIV, on the other hand, did not demonstrate independent associations with malignancy. The rigorous application of exclusion criteria enhances the internal validity of our findings, although the retrospective and single-center design limits their external validity and generalizability. However, subgroup analyses within the malignant cohort (tumor size, multifocality, lymph node metastasis, extrathyroidal extension, capsular invasion, vascular invasion, and distant metastasis) consistently yielded negative results, confirming that none of the evaluated indices demonstrated prognostic utility. This lack of prognostic relevance may be explained by the indolent course and relatively homogeneous biology of most differentiated thyroid cancers, where traditional histopathological factors (e.g., tumor size, extrathyroidal extension, and lymph node status) are more powerful predictors of outcome than systemic inflammatory markers are.

Among the three indices evaluated, the SII emerged as the most promising marker, and its statistical significance despite its weak performance may be attributed to its unique composition. A first possible explanation lies in the exclusion of monocytes from the SII formula. The SII is calculated from the neutrophil, platelet, and lymphocyte counts, while both the SIRI and PIV incorporate monocytes. This distinction is particularly relevant given the complex and context-dependent role of monocytes in tumor biology ^(27)^. In the tumor microenvironment, monocytes can differentiate into tumor-associated macrophages (TAMs), which exert both tumor-promoting and tumor-suppressive effects depending on the immune context, tumor stage, and cytokine signaling ^(27,28)^. In thyroid cancer, the influence of TAMs is less well defined and may be less dominant than in other solid tumors ^(28)^. The exclusion of monocytes from the SII may thus provide a more stable and interpretable measure of systemic inflammation relevant to thyroid tumorigenesis.

A second possible explanation relates to the broader immunological mechanisms underlying thyroid cancer. While monocyte-macrophage lineage cells play a prominent role in several malignancies, thyroid cancer pathogenesis may be more closely linked to alterations in lymphocyte subsets, such as cytotoxic T cells and natural killer (NK) cells ^(29,30)^. SII reflects not only elevated neutrophil- and platelet-driven inflammation but also a relative suppression of lymphocyte-mediated immune surveillance. A lower lymphocyte count—potentially indicating impaired NK cell or T-cell function — may correspond to reduced antitumor immunity, which is better captured by the SII. In this context, the contribution of monocytes may be less relevant, as the dominant immune dysregulation involves lymphoid and myeloid balance rather than monocyte-derived effects alone. This mechanistic difference may help explain why the diagnostic ability of the SII was superior to that of monocyte-inclusive indices such as the SIRI and PIV in our cohort ^(29,30)^.

At the molecular level, the development and progression of DTC is shaped by a delicate balance between tumor-promoting inflammation and antitumor immune responses. NK cells and cytotoxic CD8+ T lymphocytes are central to immune surveillance ^(29-31)^. However, tumors may evade immune recognition by downregulating MHC class I molecules or inducing T-cell anergy and NK cell dysfunction ^(32)^. Indeed, MHC class I loss has been documented in papillary thyroid carcinoma and is associated with reduced tumor-infiltrating lymphocytes ^(33)^. Infiltrating immune cells in the thyroid tumor microenvironment often display an exhausted phenotype, marked by the expression of immune checkpoints such as PD-1 and CTLA-4 ^(31,33)^. Additionally, tumor-derived immunosuppressive cytokines promote regulatory T-cell differentiation and inhibit effector functions ^(34)^. These mechanisms, coupled with increased neutrophil and platelet activation — which support tumor angiogenesis and metastasis—underscore the multifaceted nature of the tumor–host interaction in thyroid cancer ^(35)^. In this context, systemic inflammatory indices such as the SII may function as integrative markers that reflect the overall immune-inflammatory status of the patient.

Our findings also revealed that patients with malignant thyroid nodules were significantly younger and had higher serum TSH levels than those with benign thyroid nodules. The inverse relationship between age and malignancy is consistent with the findings of earlier studies suggesting that DTC tends to present at younger ages, potentially because of increased surveillance and earlier detection ^(36)^. Elevated TSH levels in malignant cases may be explained by the trophic effects of these hormones on thyroid follicular cells, including the promotion of proliferation and inhibition of apoptosis ^(37)^. Notably, this association remained significant even after patients with hyperthyroid conditions such as Graves’ disease and toxic multinodular goiter were excluded, supporting the notion that TSH may contribute directly to thyroid tumorigenesis, independent of functional thyroid status. Importantly, multicollinearity analyses confirmed that the SII retained its predictive significance independent of the TSH level, as the VIF values were < 2.5 and tolerance > 0.4. Moreover, in logistic regression models, the SII remained significant when it was added to a base model that included age, sex, TSH level, HbA1c level, and diabetes status, whereas the TSH level lost significance. These findings support the notion that the SII reflects an inflammatory dimension of thyroid tumorigenesis independent of the TSH level.

Despite the strengths of our study, including its structured design and stringent exclusion criteria, certain limitations must be acknowledged. The retrospective and single-center nature of the study may limit the generalizability of our findings. Another limitation is the lack of cytological data and standardized ultrasound features (such as TI-RADS), which would have provided a more comprehensive preoperative evaluation. Although glucose and HbA1c levels were included to account for the potential impact of diabetes on systemic inflammation, they were not significantly associated with malignancy in our cohort. Moreover, owing to the retrospective design, data on obesity and metabolic syndrome were not consistently available, and residual confounding by these comorbidities cannot be excluded. Finally, although all patients were scheduled for elective surgery and had no acute illness at the time of blood sampling, perioperative stress or subclinical factors within the week prior to surgery may still have influenced the systemic inflammatory indices.

From a clinical standpoint, the appeal of systemic inflammatory indices such as the SII lies not only in their diagnostic potential but also in their ease of implementation. These indices are calculated from standard complete blood count (CBC) parameters, which are routinely obtained during almost all preoperative evaluations. Unlike molecular tests or imaging modalities that may be costly or require specialized infrastructure, these indices are inexpensive and universally accessible and do not require additional procedures or add to the patient’s burden. This makes them particularly attractive for use in resource-limited settings, as well as in routine practice where high-throughput risk stratification is needed. The incorporation of the SII into preoperative assessment models may provide a low-cost adjunct to existing diagnostic strategies, although further validation is warranted.

In conclusion, while the SII emerged as an independent predictor of malignancy, its effect size was minimal, and its diagnostic accuracy was poor (AUC < 0.6). Therefore, its clinical utility should be interpreted with caution, and the SII cannot be considered a standalone diagnostic tool. Instead, it may serve as a low-cost adjunct within multivariable preoperative models, particularly in challenging scenarios such as indeterminate cytology. Future multicenter, prospective studies are necessary to validate our findings and clarify the role of systemic inflammatory markers in thyroid cancer risk assessment.

## Data Availability

datasets related to this article will be available upon request to the corresponding author.
